# Supplementary material for: Cross-Sectional Association of Dietary Patterns and Supplement Intake with Presence and Gray-Scale Median of Carotid Plaques—A Comparison between Women and Men in the Population-Based Hamburg City Health Study
Source: Nutrients. 2023 Mar 18;15(6):1468. doi: 10.3390/nu15061468 (PMC10054689; doi:10.3390/nu15061468)
Supplement: Supplementary file 1 [file nutrients-15-01468-s001.zip › nutrients-2257370-supplementary.pdf]

Supplementary Materials

Table S1. Criteria for Mediterranean Dietary Score.

Score item	MEDAS Question	Data recorded by FFQ<br>1 point given, if …
1	Do you use olive oil as the principal source of fat for cooking?	use of olive oil for the preparation of at least 2 of the following groceries: salad, vegetable, meat/fish
2	How much olive oil do you consume per day (including that used in frying, salads, meals eaten away from home, etc.)?	based on FFQ2 calculation, if >48 g vegetable oil per day
3	How many servings of vegetables do you consume per day?	based on FFQ2 calculation, if _2 portions of vegetables per day (including raw and cooked vegetables, salad,<br>olives, mushrooms except potatoes and legumes)
4	How many pieces of fruit (including fresh-squeezed juice) do you consume per day?	based on FFQ2 calculation, if _3 portions of fruit (including fruit, mixed fruit, fruit salad, mixed stewed fruit and fruit juices excluding sweetened beverages)
5	How many servings of red meat, hamburger, or sausages do you consume per day?	based on FFQ2 calculation, if <100 g red meat (eg beef, veal, pork, lamb) and processed meat products
6	How many servings (12 g) of butter, margarine, or cream do you consume per day?	based on FFQ2 calculation, if <1 portion butter, margarine and cream and other animal fat
7	How many carbonated and/or sugar-sweetened<br>beverages do you consume per day?	based on FFQ2 calculation, sugar-sweetened beverages <1 portion per day (including lemonade and colas)
8	Do you drink wine? How much do you consume<br>per week?	based on FFQ2 calculation, if _7 portions wine (red and white wine; 1 portion = 0.251)
9	How many servings of pulses do you consume per week?	_3 portions pulses (e.g., beans, lentils, peas, chickpeas)
10	How many servings of fish/seafood do you consume per week?	based on FFQ2 calculation, if _3 portions fish, fish<br>products and seafood per week

11	How many times do you consume commercial (not homemade) pastry such as cookies or cake per week?	based on FFQ2 calculation, if <3 portions cakes, chocolate, cookies, sweets with and without chocolate per week
12	How many times do you consume nuts per week?	based on FFQ2 calculation, if _3 portions nuts per week
13	Do you prefer to eat chicken, turkey or rabbit instead of beef, pork, hamburgers, or sausages?	Based on FFQ2 calculation, if g white meat (e.g., chicken, hen and other poultry) > g red meat (e.g., beef, veal, pork, lamb and processed meat products)
14	How many times per week do you consume boiled vegetables, pasta, rice, or other dishes with a sauce of tomato, garlic, onion, or leeks sautéed in olive oil?	>1–2 times a week tomato sauce

Table S2. Scoring system for Dietary Approaches to Stop Hypertension (DASH) diet.

Score item	DASH component	Scoring
	Total Grain	
1		
	≥7 servings/day	1
	5–6 servings/day	0.5
	<5 servings/day	0
2	Vegetables	1
	≥4 servings/day	0.5
	2–3 servings/day	0
	<2 servings/day	
3	Fruits	1
	≥4 servings/day	0.5
	2–3 servings/day	0
	<2 servings/day	

4	Total dairy	1
	≥2 servings/day	0.5
	1 servings/day	0
	<1 serving/day	
5	Meat, poultry, and fish	1
	≥2 servings/day	0.5
	3 servings/day	0
	≥4 serving/day	
6	Nuts, seeds, and legumes	1
	≥4 servings/day	0.5
	2–3 servings/day	0
	<2 servings/day	
7	% kcal from fat	1
	≤27%	0.5
	≥28 ≤29%	0
	≥30%	
8	% kcal from saturated fat	1
	≤6%	0.5
	≤7 ≥8%	0
	≥9%	
9	Sweets	1
	≤5 servings/week	0.5
	6–7 servings/week	0
	≥8 serving/week	
10	Sodium	1
	≤2400 mg/day 1	0.5
	2400–3000 mg/day 0.5	0
	>3000 mg/day	

Table S3. Logistic regression models regarding the presence of carotid plaques.

Characteristics	MEDAS		DASH		Supplement intake	
	OR (95% CI)	*p*-value	OR (95% CI)	*p*-value	OR (95% CI)	*p*-value
Age 66-77 vs. age ≤ 65	2.53 (2.23, 2.87)	<0.001	2.5 (2.21, 2.84)	<0.001	2.56 (2.25, 2.9)	<0.001
Male vs. female sex	1.61 (1.41, 1.84)	<0.001	1.6 (1.41, 1.82)	<0.001	1.52 (1.34, 1.73)	<0.001
Low vs. medium education	1.22 (1.03, 1.45)	0.02	1.22 (1.03, 1.45)	0.023	1.21 (1.02, 1.44)	0.03
High vs. medium education	0.93 (0.79, 1.1)	0.400	0.93 (0.79, 1.1)	0.4	0.95 (0.8, 1.13)	0.571
BMI	0.99 (0.97, 1)	0.065	0.99 (0.97, 1)	0.078	0.99 (0.97, 1)	0.075
Diabetes yes vs. no	1.09 (0.87, 1.35)	0.449	1.09 (0.88, 1.35)	0.447	1.1 (0.88, 1.36)	0.415
Hypertension yes vs. no	1.72 (1.49, 1.99)	<0.001	1.72 (1.49, 1.99)	<0.001	1.69 (1.47, 1.96)	<0.001
Hyperlipidemia yes vs. no	1.69 (1.45, 1.97)	<0.001	1.68 (1.45, 1.96)	<0.001	1.7 (1.45, 1.98)	<0.001
Current smoking vs. Non-smoking	1.7 (1.46, 1.98)	<0.001	1.7 (1.46, 1.98)	<0.001	1.67 (1.44, 1.94)	<0.001
Metabolic syndrome yes vs. no	1.15 (0.98, 1.34)	0.076	1.15 (0.99, 1.34)	0.075	1.14 (0.97, 1.33)	0.104
Heart failure yes vs. no	1.22 (0.92, 1.62)	0.166	1.22 (0.92, 1.62)	0.176	1.24 (0.93, 1.65)	0.138
Atrial fibrillation yes vs. no	1.08 (0.85, 1.37)	0.524	1.09 (0.86, 1.38)	0.489	1.09 (0.86, 1.39)	0.463
Myocardial infarction ever vs. never	1.34 (0.95, 1.91)	0.101	1.35 (0.95, 1.92)	0.093	1.34 (0.95, 1.91)	0.095
Stroke ever vs. never	1.32 (0.95, 1.84)	0.096	1.34 (0.96, 1.86)	0.084	1.29 (0.93, 1.79)	0.126
Regular physical exercise yes vs. no	0.84 (0.69, 1.03)	0.095	0.85 (0.69, 1.04)	0.114	0.86 (0.71, 1.06)	0.153

This table shows results of additional analyses of logistic regression models regarding the presence of carotid plaques in full HCHS-sub-cohort, including 10,000 participants. All models are adjusted for age, sex, education, body-mass index, diabetes mellitus, arterial hypertension, hyperlipidemia, smoking status, heart failure, atrial fibrillation, myocardial infarction, stroke and sports. Abbreviations: OR, odds ratio; CI, confidence interval; MEDAS, Mediterranean Diet Adherence Score; DASH, Dietary Approach to Stop Hypertension
